# Genomic Diversity and Antimicrobial Resistance of Haemophilus Colonizing the Airways of Young Children with Cystic Fibrosis

**DOI:** 10.1128/msystems.00178-21

**Published:** 2021-08-31

**Authors:** Stephen C. Watts, Louise M. Judd, Rosemary Carzino, Sarath Ranganathan, Kathryn E. Holt

**Affiliations:** a Department of Biochemistry and Molecular Biology, Bio21 Molecular Science and Biotechnology Institute, University of Melbourne, Melbourne, Victoria, Australia; b Department of Infectious Diseases, Central Clinical School, Monash University, Melbourne, Victoria, Australia; c Infection and Immunity, Murdoch Children’s Research Institute, Melbourne, Victoria, Australia; d Department of Paediatrics, University of Melbourne, Melbourne, Victoria, Australia; e London School of Hygiene & Tropical Medicine, London, United Kingdom; University of California, Irvine

**Keywords:** *Haemophilus*, *Haemophilus influenzae*, *Haemophilus parainfluenzae*, antibiotic resistance, cystic fibrosis, genomics

## Abstract

Respiratory infection during childhood is a key risk factor in early cystic fibrosis (CF) lung disease progression. Haemophilus influenzae and Haemophilus parainfluenzae are routinely isolated from the lungs of children with CF; however, little is known about the frequency and characteristics of Haemophilus colonization in this context. Here, we describe the detection, antimicrobial resistance (AMR), and genome sequencing of H. influenzae and *H. parainfluenzae* isolated from airway samples of 147 participants aged ≤12 years enrolled in the Australian Respiratory Early Surveillance Team for Cystic Fibrosis (AREST CF) program, Melbourne, Australia. The frequency of colonization per visit was 4.6% for H. influenzae and 32.1% for *H. parainfluenzae*, 80.3% of participants had H. influenzae and/or *H. parainfluenzae* detected on at least one visit, and using genomic data, we estimate 15.6% of participants had persistent colonization with the same strain for at least two consecutive visits. Isolates were genetically diverse and AMR was common, with 52% of H. influenzae and 82% of *H. parainfluenzae* displaying resistance to at least one drug. The genetic basis for AMR could be identified in most cases; putative novel determinants include a new plasmid encoding *bla*_TEM-1_ (ampicillin resistance), a new inhibitor-resistant *bla*_TEM_ allele (augmentin resistance), and previously unreported mutations in chromosomally carried genes (*pbp3*, ampicillin resistance; *folA*/*folP*, cotrimoxazole resistance; *rpoB*, rifampicin resistance). Acquired AMR genes were more common in *H. parainfluenzae* than H. influenzae (51% versus 21%, *P* = 0.0107) and were mostly associated with the ICE*Hin* mobile element carrying *bla*_TEM-1_, resulting in more ampicillin resistance in *H. parainfluenzae* (73% versus 30%, *P* = 0.0004). Genomic data identified six potential instances of Haemophilus transmission between participants, of which three involved participants who shared clinic visit days.

**IMPORTANCE** Cystic fibrosis (CF) lung disease begins during infancy, and acute respiratory infections increase the risk of early disease development and progression. Microbes involved in advanced stages of CF are well characterized, but less is known about early respiratory colonizers. We report the population dynamics and genomic determinants of AMR in two early colonizer species, namely, Haemophilus influenzae and Haemophilus parainfluenzae, collected from a pediatric CF cohort. This investigation also reveals that *H. parainfluenzae* has a high frequency of AMR carried on mobile elements that may act as a potential reservoir for the emergence and spread of AMR to H. influenzae, which has greater clinical significance as a respiratory pathogen in children. This study provides insight into the evolution of AMR and the colonization of H. influenzae and *H. parainfluenzae* in a pediatric CF cohort, which will help inform future treatment.

## INTRODUCTION

Cystic fibrosis (CF) is a common inherited genetic disorder caused by deleterious mutations in the cystic fibrosis transmembrane conductance regulator gene (1). Although the disease is multisystemic, the primary cause of morbidity and mortality results from pulmonary dysfunction. CF lung disease manifests as delayed mucociliary clearance and mucus adhesion leading to recurrent and chronic microbial infections (2), which elicit an adverse host inflammation response resulting in bronchiectasis and ultimately respiratory failure (3, 4). Management of bacterial lung infections is essential in CF disease trajectory and can be managed in part through antimicrobial therapy. However, antimicrobial resistance (AMR) is frequently acquired through various mechanisms and can have clinical consequences in CF patients, including reduced lung function (5–7).

There are a small number of bacterial species that predominantly cause CF lung infections, including Pseudomonas aeruginosa, Burkholderia cepacia complex, Staphylococcus aureus, Stenotrophomonas maltophilia, and Haemophilus influenzae (8). Importantly, acute respiratory infection in newborns with CF is an established risk factor for early disease development and progression (9).

H. influenzae and Haemophilus parainfluenzae are among the most common Haemophilus species that colonize the respiratory tract of children in early life (10, 11). H. influenzae is considered an opportunistic pathogen and can cause invasive disease. Instances of invasive *H. parainfluenzae* infection have also been described (12–14); however, *H. parainfluenzae*-related disease is less frequently observed, and *H. parainfluenzae* is recognized as having a lower pathogenic capacity than H. influenzae. Both H. influenzae and *H. parainfluenzae* are routinely isolated from the respiratory tract of children with CF, particularly during episodes of disease exacerbation (15). Although many of the classical pathogens involved in CF lung disease have been well studied, less is known about the role of Haemophilus species during the critical period of early childhood. Such knowledge is essential, as it is increasingly recognized that CF lung disease commences soon after diagnosis in early infancy and progresses thereafter (16, 17). Further insight into the epidemiology and resistance profiles of these early colonizing and infecting bacteria will inform future treatment practices.

The emergence and accumulation of AMR in H. influenzae and *H. parainfluenzae* is common, with the highest resistance rates reported for ampicillin (AMP; 23.9 to 58.5% in H. influenzae, 13.2 to 50.0% in *H. parainfluenzae*) and cotrimoxazole (21.4 to 71.1% in H. influenzae, 14.9 to 44.2% in *H. parainfluenzae*) (18–31). Generally, H. influenzae infections are treated with β-lactams, such as extended-spectrum penicillins or cephalosporins (32). Other drugs are often used in combination with or as an alternative to β-lactams and include antifolates, quinolones, and macrolides. Resistance to these drugs typically arises through either acquisition of horizontally transferred resistance genes or mutations in chromosomally encoded protein targets (32). Acquired AMR genes in H. influenzae and *H. parainfluenzae* are frequently localized within mobile genetic elements, such as ICE*Hin* or small plasmids (33–35), which appear to have facilitated the emergence of multidrug-resistant H. influenzae and *H. parainfluenzae* strains in recent years (36, 37).

H. influenzae is known to produce a polysaccharide capsule, which can be classified into six serotypes (Hia through Hif) and is an invasive virulence determinant (38). Strains that do not produce the capsule are designated nontypeable H. influenzae (NTHi). The introduction of the highly effective Hib conjugate vaccines caused a marked reduction of Hib-related disease incidence but consequently resulted in an increased prevalence of NTHi-related disease (39); NTHi is now more commonly isolated from children with CF than any encapsulated H. influenzae serotype (40, 41). *H. parainfluenzae* is generally less well characterized, and the role it may have in CF disease is unclear. There is no detailed description of encapsulated *H. parainfluenzae*, although there is increasing evidence that some *H. parainfluenzae* strains could express a polysaccharide capsule (37). Moreover, there is a stark lack of *H. parainfluenzae* genomic data compared with H. influenzae, despite that it occupies a similar niche. Here, we investigate the prevalence, genomic diversity, and AMR phenotypes of H. influenzae and *H. parainfluenzae* colonizing the airways of children with CF, recruited at the Royal Children’s Hospital (RCH), Melbourne, Australia.

## RESULTS

### Detection and sequencing of Haemophilus isolates.

During the 1-year study period, 147 AREST CF study participants receiving treatment at the RCH CF specialist clinic were screened for the presence of H. influenzae or *H. parainfluenzae* in the respiratory tract during regular clinic visits and during hospitalization for pulmonary exacerbation. Participant characteristics are given in Table 1. Thirty (20.4%) participants tested culture positive for colonization with H. influenzae in ≥1 sample and 111 (75.5%) participants for *H. parainfluenzae*. Only 29 participants (19.7%) had no Haemophilus-positive samples; these individuals did not differ by age or gender but contributed fewer samples each (mean 4.6 versus 6.0, *P* = 0.012 using two-sample Kolmogorov-Smirnov test). The overall frequency of colonization per visit was 4.6% for H. influenzae and 32.1% for *H. parainfluenzae*, with 86 participants (58.5%) presenting with either H. influenzae or *H. parainfluenzae* on 2 or more occasions. Several participants had both H. influenzae and *H. parainfluenzae* detected during the same (*n* = 10, 6.8%) or different (*n* = 15, 10.2%) visits.

**TABLE 1:** Study participant characteristics

Characteristic	Data for:	
	All participants (lab-reported species identification)	Participants with WGS data (WGS species identification)
All participants		
No. of total participants	147	59
No. of females (%)	64 (43.5)	23 (38.9)
Mean age at first sample, yrs (range)	5.7 (0.08–11.8)	4.1 (0.10–8.9)
Mean no. of samples, count (range)	5.8 (1–12)	6.2 (2–12)
No. with ≥1 H. influenzae-positive sample (%)	30 (20.4)	21 (35.6)
No. with ≥1 *H. parainfluenzae*-positive sample (%)	111 (75.5)	55 (93.2)
No. with Zero H. influenzae*-* or *H. parainfluenzae*-positive samples (%)	29 (19.7)	
Participants with ≥1 Haemophilus culture-positive sample		
No. of total participants	118	59
No. of females (%)	54 (45.8)	23 (39.0)
Mean age at first sample, yrs (range)	5.3 (0.08–11.8)	4.1 (0.10–8.9)
Mean no. of samples (range)	6.0 (2–12)	6.2 (2–12)
Mean no. of H. influenzae-positive samples (range)	0.3 (0–3)	0.5 (0–3)
Mean no. of *H. parainfluenzae*-positive samples (range)	2.3 (0–7)	2.8 (0–7)

A subset of 162 Haemophilus isolates from 59 participants (50% of culture-positive individuals, representative in terms of age and gender) (see Table 1) were subjected to whole-genome sequencing (WGS), of which 89.5% were sequenced successfully. WGS data revealed some species misidentifications (5.5%) and mixed cultures (13%), leaving 24 H. influenzae and *H. parainfluenzae* isolates for further analysis (see Fig. S1 online at doi.org/10.26188/14954931 and Materials and Methods).

### Genetic diversity and population structure.

**FIG 1:**
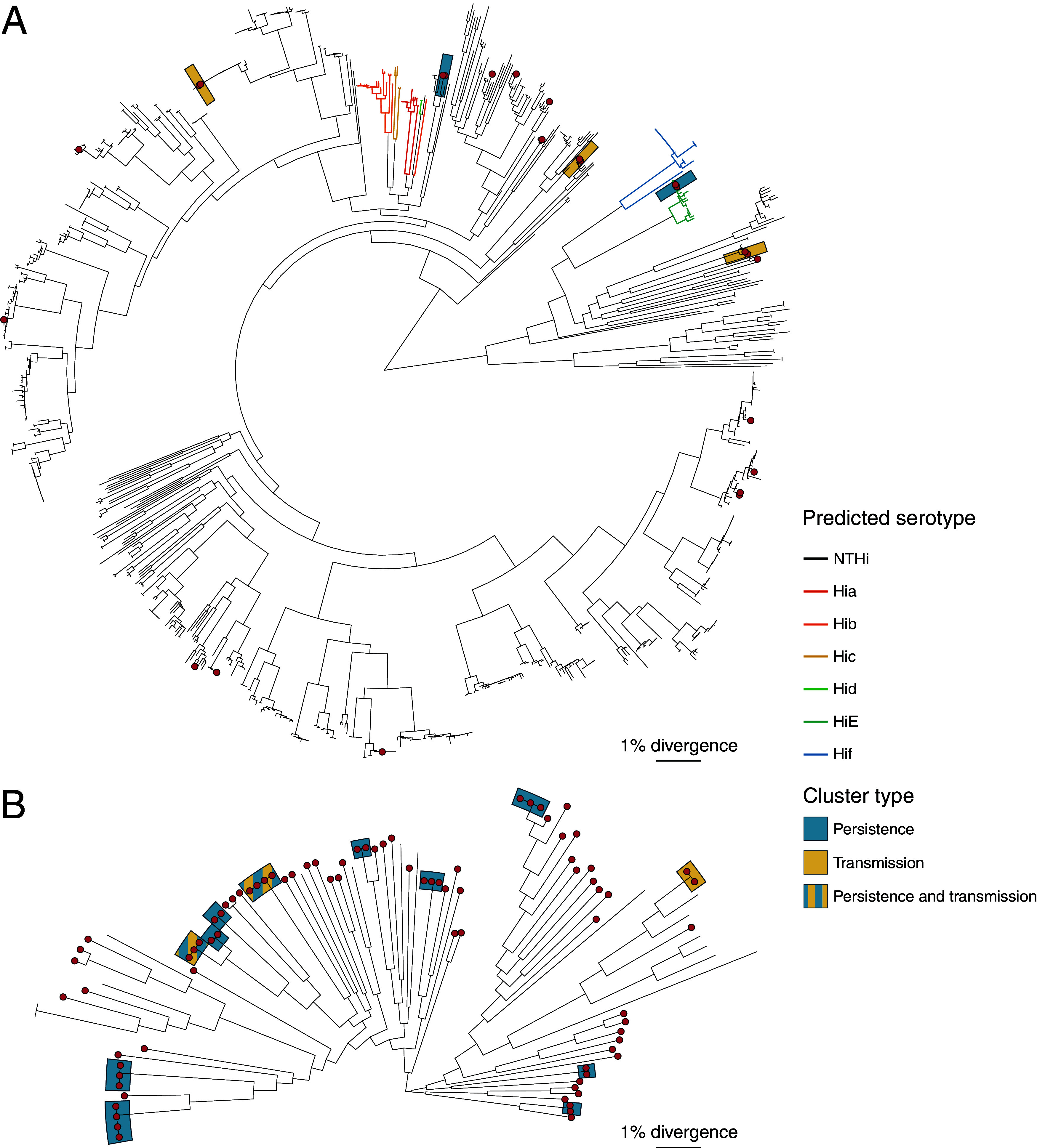
Maximum likelihood phylogenies for H. influenzae (A) and *H. parainfluenzae* (B). Trees were inferred from alignments of core genome SNVs, showing the relationship between RCH isolates (red tips) and publicly available genome collections (summarized in Table S1 online at doi.org/10.26188/14954931). Shading indicates strain clusters of RCH isolates involved in potential transmission or persistence (see Fig. 4).

The H. influenzae isolates collected in this study were highly diverse (median of 2.3% nucleotide divergence in conserved core genome), and a comparison with publicly available WGS data from other studies (*n* = 877 genomes, see Data Set S1 in the supplemental material) indicates RCH CF isolates are distributed across the global species phylogeny (red tips in Fig. 1A). All but two RCH CF H. influenzae genomes had no detectable capsule biosynthesis (*cap*) locus and fell outside the small number of lineages typically associated with encapsulation (42) (colored branches in Fig. 1A); they are thus predicted to be unencapsulated or nontypeable. Two RCH CF isolates (drug susceptible, sequence type 18 [ST18], from the same patient) carried intact copies of the *cap*-e locus and fell within the lineage typically associated with serotype e (green branches in Fig. 1A); thus, they are predicted to express serotype e capsules.

10.1128/mSystems.00178-21.1 DATA SET S1 Isolates sequenced in this study. Download Data Set S1, XLSX file, 0.1 MB. Copyright © 2021 Watts et al. 2021 Watts et al. https://creativecommons.org/licenses/by/4.0/ This content is distributed under the terms of the Creative Commons Attribution 4.0 International license.

**FIG 2:**
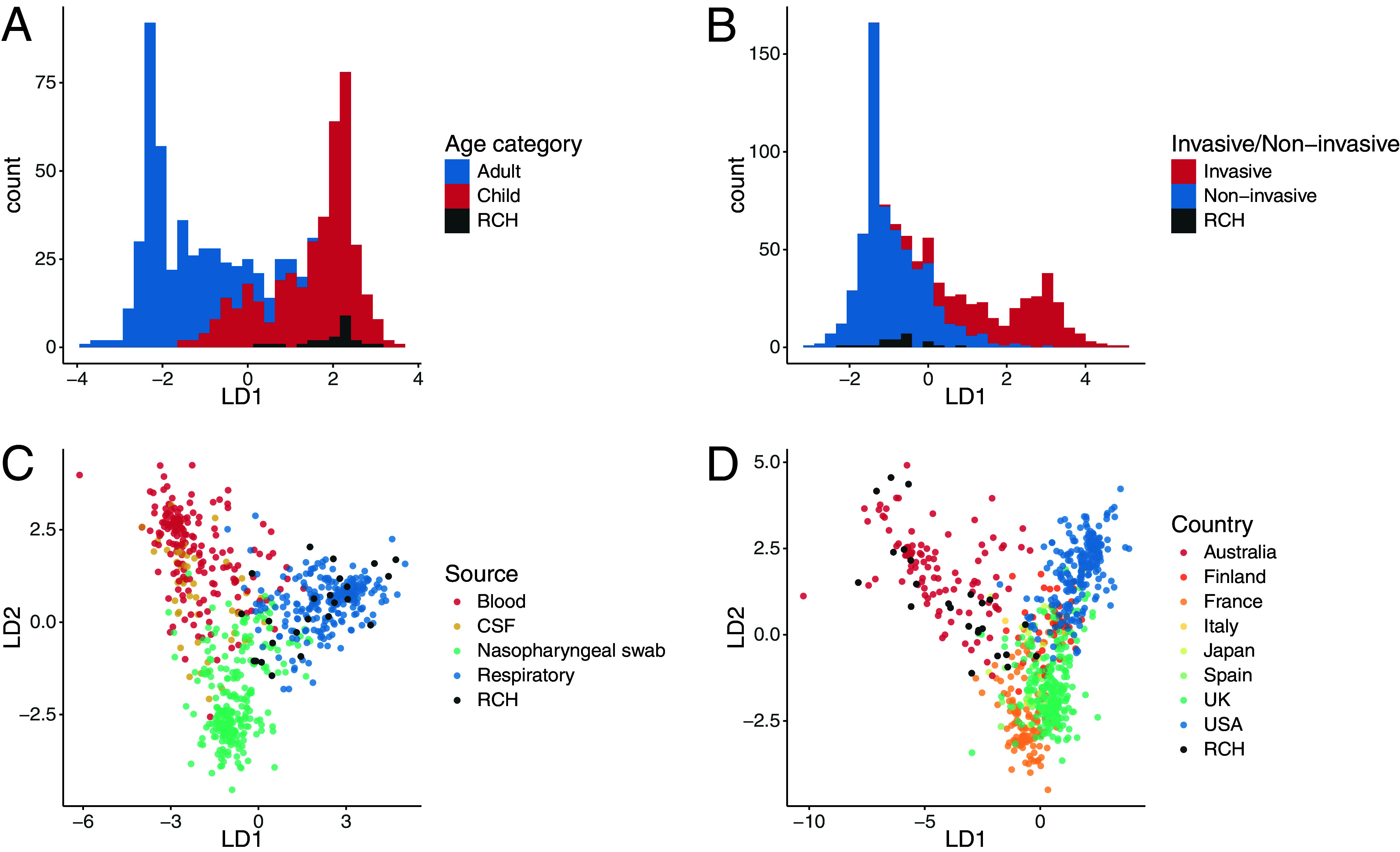
Discriminant analysis of principal components for H. influenzae isolates. Analyses were based on k-mer profiles extracted from the genomes of novel Australian CF pediatric respiratory colonizing isolates sequenced for this study (labeled RCH, black) and those available in public genome collections (other colors; data sources are summarized in Data Set S1). Plots show the distribution of values for the significant linear discriminants (LD1 and LD2) included in the linear discriminant functions, which were constructed to discriminate genomes on the basis of host age (A), infection status (invasive or noninvasive/colonizing) (B), specimen type (C), and country of isolation (D).

We used discriminant analysis of principal components (DAPC) to explore how the population of RCH CF H. influenzae isolates compared with the global H. influenzae population structure captured by k-mer profiles of publicly available WGS data from a range of other contexts (see Data Set S1; see Fig. S4 online at doi.org/10.26188/14954931). The data indicate that our Australian pediatric CF isolates are typical of noninvasive respiratory isolates from children in other settings, based on functions constructed to discriminate these parameters from isolates collected from adults, nonrespiratory specimens, and invasive disease (Fig. 2A to C). RCH isolates clustered most closely with other Australian isolates in the discriminant function based on geographical location (Fig. 2D). Notably, DAPC analysis of the 264,940 core-genome single nucleotide variants (SNVs) used for phylogenetic inference yielded much weaker discriminant functions for specimen type and geographical location (see Fig. S5 online at doi.org/10.26188/14954931), suggesting that the discriminant genetic features are carried in the accessory genome rather than core gene allelic variation.

The *H. parainfluenzae* isolates collected in this study also show extensive genetic diversity (median 5.1% nucleotide divergence in conserved core genome). This finding is harder to contextualize within the overall species diversity due to the low number of genomes available from other studies but appears to be representative of species-wide phylogenetic diversity (Fig. 1B).

### Antimicrobial resistance.

AMR was relatively common, with 52% of H. influenzae and 82% of *H. parainfluenzae* isolates displaying resistance to at least one of the five drugs tested (Table 2). The frequency of cotrimoxazole (STX) resistance was similar in both species (35% in H. influenzae, 31% in *H. parainfluenzae*), but ampicillin and/or augmentin (AMC) resistance was observed at significantly higher rates in *H. parainfluenzae* than those in H. influenzae (*P < *0.05) (see Table 2). Rifampicin (RIF) was observed at low frequencies in both species (17% in H. influenzae, 7% in *H. parainfluenzae*). Multidrug resistance (defined here as resistance to ampicillin or augmentin plus at least one other drug class) was also more commonly detected in *H. parainfluenzae*, although the difference was not statistically significant (see Table 2).

**TABLE 2:** Frequency of nonsusceptibility to antimicrobials among sequenced isolates^*a*^

Antimicrobial	No. (%) positive for:		Odds ratio (95% CI)	*P* value
	H. influenzae	*H. parainfluenzae*		
Ampicillin	7/23 (30)	60/82 (73)	6.11 (2.05–20.09)	0.0004
Augmentin	1/17 (6)	25/79 (32)	7.30 (1.02–322.38)	0.0351
Cefotaxime	0/23 (0)	2/81 (3)	Inf (0.053–Inf)	1
Cotrimoxazole	8/23 (35)	25/82 (31)	0.82 (0.28–2.55)	0.8002
Rifampicin	2/12 (17)	4/59 (7)	0.37 (0.045–4.61)	0.2659
Ampicillin or augmentin	7/17 (41)	62/78 (80)	5.41 (1.58–19.72)	0.0026
One or more drugs tested	12/23 (52)	68/83 (82)	4.09 (1.36–12.47)	0.0058
MDR	5/23 (22)	23/83 (28)	1.38 (0.42–5.30)	0.7897

a Nonsusceptibility was defined as I or R according to clinical breakpoints (see Materials and Methods). Multidrug resistance (MDR) was defined as resistance to ampicillin or augmentin plus at least one other antimicrobial. Association tests compare resistance rate between species and were performed using Fisher’s exact test. The analysis is restricted to isolates that were successfully sequenced, had a susceptibility phenotype reported for the given antimicrobial(s), and found to be pure cultures, with species identification based on genome data. Inf, infinity.

**FIG 3:**
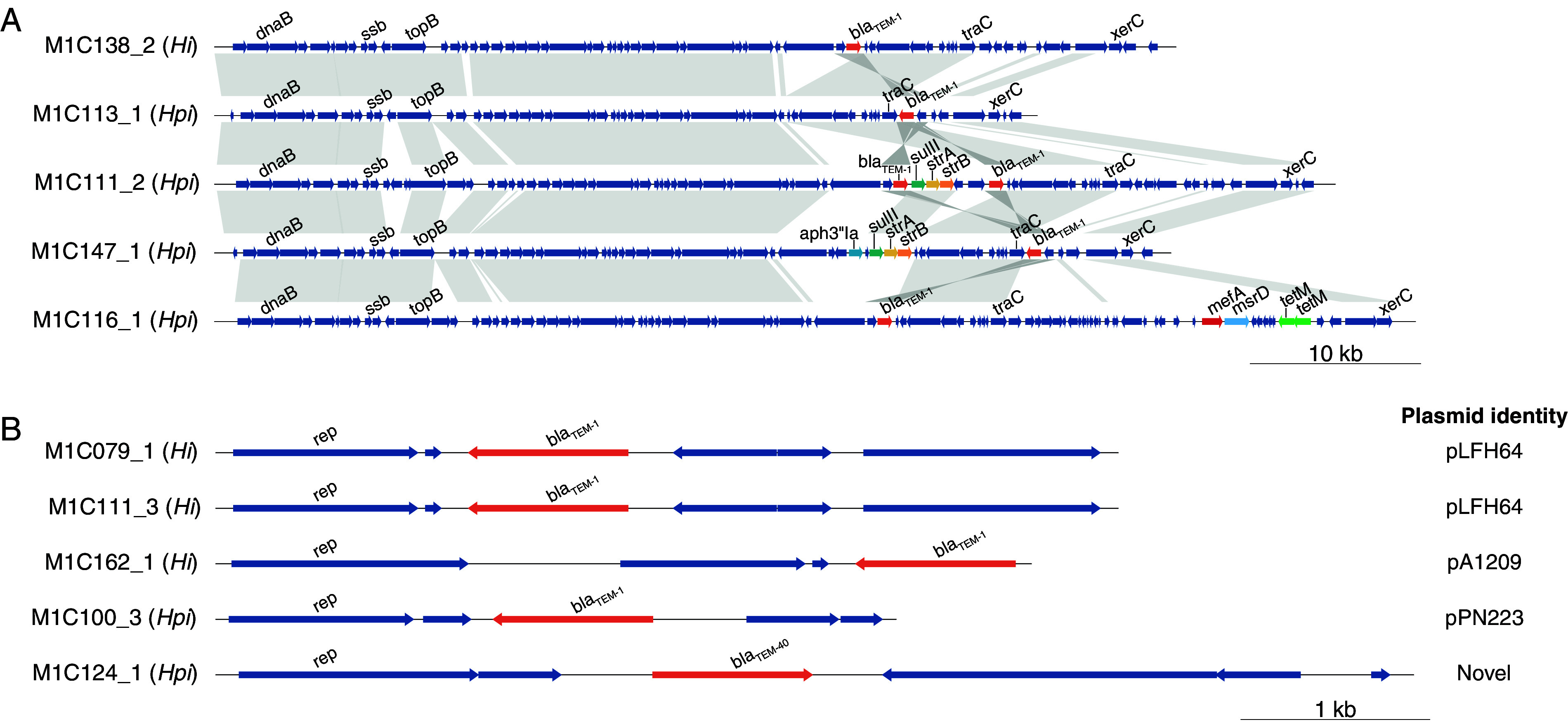
Representative ICE*Hin* structures (A) and plasmids (B) carrying AMR genes identified in RCH isolates. Plasmids are annotated with the best corresponding match in the NCBI nucleotide database, see Table S3 online at doi.org/10.26188/14954931.

We used the WGS data to explore genetic determinants of AMR in the RCH isolates. Horizontally acquired AMR genes were more frequently found in *H. parainfluenzae* than in H. influenzae, with 42 (51%) *H. parainfluenzae* and 5 (21%) H. influenzae isolates containing one or more acquired AMR genes (*P* = 0.0107 using Fisher’s exact test) (see DATA SET S2 in the supplemental material). Most common were *bla*_TEM_ genes (36 *bla*_TEM-1_, 4 *bla*_TEM-40_, and 2 *bla*_TEM-30_), carried in the mobile element ICE*Hin* (2 H. influenzae, 38 *H. parainfluenzae*) or small plasmids (3 H. influenzae, 2 *H. parainfluenzae*). Other AMR genes were less common and restricted to *H. parainfluenzae*, which were generally located in ICE*Hin* elements (11 isolates carried *strAB* [aminoglycosides] and *sul1* [sulfonamide-cotrimoxazole], 5 *aph3’la* [aminoglycosides], 1 *tetB* [tetracycline], and 1 *tetM* [tetracycline] with *msrD* and *mefA* [macrolides]). The resistance cassettes of ICE*Hin* varied in structure and gene content (Fig. 3A). Four distinct *bla*_TEM_ plasmids were observed and were of a similar size (4.3 to 6.5 kbp). Three plasmids were homologous to previously sequenced H. influenzae plasmids pLFH64 (2 H. influenzae, *bla*_TEM-1_), pA1209 (one H. influenzae, *bla*_TEM-1_), pPN223 (one *H. parainfluenzae*, *bla*_TEM-1_); the fourth was a novel plasmid present in one *H. parainfluenzae* isolate (pM1C124_1, *bla*_TEM-40_; deposited in GenBank under accession MW111541) (Fig. 3B; see Table S4 online at doi.org/10.26188/14954931). Acquired AMR genes accounted for only 33.6% of observed nonsusceptibility phenotypes; hence, we screened for mutations in conserved core resistance-related chromosomal genes that could potentially explain resistance to ampicillin and augmentin (*pbp* genes) (see Table S3 online at doi.org/10.26188/14954931), cotrimoxazole (*folP* and *folA*), and rifampicin (*rpoB*) (summarized in Tables S5 and S6 at doi.org/10.26188/14954931).

10.1128/mSystems.00178-21.2 DATA SET S2 AMR phenotypes of isolates and genetic mechanisms of resistance. Download Data Set S2, XLSX file, 0.03 MB. Copyright © 2021 Watts et al. 2021 Watts et al. https://creativecommons.org/licenses/by/4.0/ This content is distributed under the terms of the Creative Commons Attribution 4.0 International license.

Ampicillin resistance in H. influenzae could be entirely explained by acquired β-lactamases encoded by *bla*_TEM_ genes (57%) and/or mutations in the penicillin-binding proteins PBP3 (FtsI) and PBP1B (MrcB) (Table S5 online at doi.org/10.26188/14954931). In *H. parainfluenzae*, acquired *bla*_TEM_ could explain 60% of ampicillin resistance, but we detected no known or novel PBP mutation that was statistically associated with resistance (Table S6 online at doi.org/10.26188/14954931). Notably though, the first *H. parainfluenzae* isolated from participant M1C152 was ampicillin sensitive and wild type at PBP3-502. The two subsequent *H. parainfluenzae* isolates from this participant (following treatment with augmentin and ceftriaxone) were ampicillin resistant with no acquired AMR genes and differed from the first by a single SNV across the entire genome resulting in the amino acid substitution PBP3-A502T, supporting the previously reported role of this mutation in conferring resistance (43). Nevertheless, our findings leave 33% of ampicillin resistance in *H. parainfluenzae* unexplained.

Augmentin is a combination of amoxicillin plus the β-lactamase inhibitor clavulanic acid. Just four augmentin-resistant isolates (16%) carried inhibitor-resistant β-lactamase alleles (2 *H. parainfluenzae* with *bla*_TEM-30_, 2 *H. parainfluenzae* with *bla*_TEM-40_). Nine more isolates (36%) carried *bla*_TEM-1_, but this encoded β-lactamase is susceptible to clavulanic acid inhibition and we identified no *pbp* variants in these isolates that were significantly associated with augmentin resistance; hence, inhibitor resistance remains unexplained in these cases. Two augmentin-resistant *H. parainfluenzae* isolates collected from the same participant (M1C141) contained novel *bla*_TEM_ alleles that share substitution mutations with known inhibitor-resistant alleles (*bla*_TEM-1_-M67I, *bla*_TEM-1_-W163L) (Fig. S6 online at doi.org/10.26188/14954931), which likely explain the phenotype (44, 45). Hence, the vast majority of augmentin resistance (100% in H. influenzae, 80% in *H. parainfluenzae*) is unexplained. Resistance to the third-generation cephalosporin cefotaxime (CTX) was observed in two *H. parainfluenzae* isolates from different participants but was unexplained (one carried no acquired genes, one carried only *bla*_TEM-1_, and neither carried unique *pbp* mutations).

Cotrimoxazole is a combination of trimethoprim and sulfamethoxazole. Resistance to trimethoprim is associated with mutations in the chromosomal dihydrofolate reductase *folA* or acquisition of mobile resistant alleles (*dfr* genes), while resistance to sulfamethoxazole requires mutations in the chromosomal dihydropteroate synthase *folP* or acquisition of mobile resistant alleles (*sul* genes). In H. influenzae, no acquired *sul* or *dfr* genes were detected; however, all cotrimoxazole-resistant isolates carried a novel resistance-associated mutation, FolA-N13S, and most carried the novel FolP-G189C (75%), as well as previously reported FolA-I95L (75%) and FolP-P64ins (38%) (Table S5 online at doi.org/10.26188/14954931, and Data Set S2). In *H. parainfluenzae*, 92% of cotrimoxazole-resistant isolates carried *sul1* (36%) and/or resistance-associated FolP mutations (including FolP-P64ins and FolP-G189C, 80%); 64% carried resistance-associated FolA mutations (46) (Table S6 online at doi.org/10.26188/14954931, and Data Set S2).

Rifampicin resistance is most often explained by mutations in *rpoB* (the RNA polymerase beta subunit), including one previous report in H. influenzae (47). Both rifampicin-resistant H. influenzae isolates (from the same patient, M1C073) carried a novel mutation, RpoB-A1131T, that was absent from sensitive isolates. In *H. parainfluenzae*, one of four rifampicin-resistant isolates (M1C081_2) carried RpoB-T724I, which has been previously described in resistant *H. parainfluenzae* isolates (19); however, the remaining three isolates contained no other mutations associated with rifampicin resistance (Data Set S2).

### Persistent colonization and transmission.

**FIG 4:**
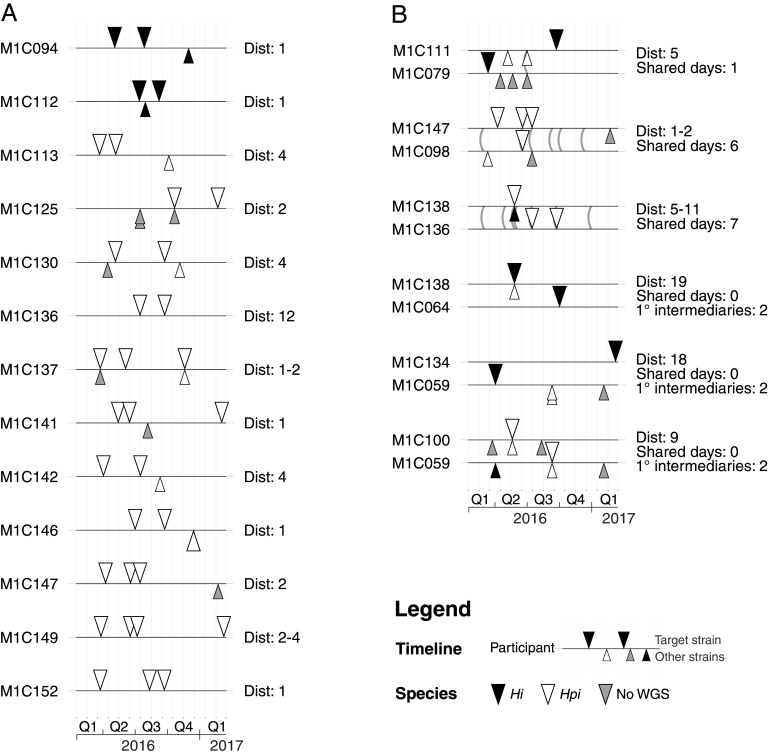
Timelines of Haemophilus isolation for participants affected by persistent colonization (A) or potential transmission between participants (B). Triangles indicate Haemophilus isolates that are colored according to WGS-confirmed species as per inset legend. Isolates presented above each timeline represent the same strain (defined as pairwise genetic distance <20; see Materials and Methods) and those below are different strains. Dist, range of pairwise genetic distances (nonrecombinant SNVs + number of inferred recombination events) observed between isolates of the same strain. In B, lines connecting participant timelines represent instances where participants attended an RCH clinic during the same day. Shared days, number of days on which both participants attended the RCH CF clinic; 1° intermediaries, for participant pairs not sharing any clinic visit days with one another, we searched for primary intermediary participants who shared at least one clinic data point with each of the participants.

Seventy-nine participants (53.7%) were culture positive for the same Haemophilus species on ≥1 occasion; 7 (4.8%) participants had ≥2 H. influenzae and 75 (51%) had ≥2 *H. parainfluenzae*. The probability of testing culture positive for the same species in the next sample after an initial positive result (mean time interval 105 days) was 16.0% for H. influenzae and 48.1% for *H. parainfluenzae* (*P* = 0.004 for test of difference in proportions). Among those individuals who had a culture-positive sample directly followed by a culture-negative for the same species (*n* = 107), 36 (33.6%) had a subsequent positive sample and 11 (10.3%) had no further samples tested. In 45/79 participants, WGS data were available for at least 2 isolates of the same species. Among these patients with ≥2 WGS sequences, 13 participants (29%) had matching isolates of the same strain (defined as ≤20 mutations; see Materials and Methods), consistent with persistent colonization (2 H. influenzae and 11 *H. parainfluenzae*) (see Fig. 4A). Assuming the same rate of strain matching (29%) among the 34 participants who had ≥2 isolates but WGS data was available for only 1 of those isolates, we estimate that a further 10 of these participants would have matching strains. Thus, we estimate a total of 23 (15.6%) of the 147 participants (95% confidence interval [CI], 8.4% to 22.6%) had Haemophilus colonization that persisted between visits. Notably, the only encapsulated strain detected in this study (cap-e H. influenzae) was detected twice in the same participant (M1C094), on two separate clinic visits 84 days apart.

WGS data showed that most strains were unique to a single participant; however, we identified 6 participant pairs that shared the same strain (1 to 19 mutations, see Materials and Methods; 3 H. influenzae, 3 *H. parainfluenzae*) (see Fig. 4B), suggesting potential transmission. In three such cases, both participants visited an RCH CF clinic on the same day (Fig. 4B), providing a potential opportunity for transmission within the clinic. A contact network reconstructed from visit dates showed that while the other three strain-sharing participant pairs never attended on the same day, they did have shared visit days with single-step intermediary participants (Fig. S7 online at doi.org/10.26188/14954931); hence, it is possible that one of these intermediary participants had become colonized at the clinic via the first participant and then passed the strain on to the second participant on a subsequent visit.

Twelve of the 13 (92%) WGS-confirmed cases of persistent strain colonization exhibited resistance to at least 1 drug, compared with 70% of strains that were not identified as persisting (see Table S7 online at doi.org/10.26188/14954931) (Data Set S2). For *H. parainfluenzae*, all AMR phenotypes were more frequent among isolates associated with persistent strain colonization; however, these comparisons were underpowered, and the differences were only statistically significant for ampicillin, augmentin, and cefotaxime (Table S7 at doi.org/10.26188/14954931). Five of the six potentially transmitted strains displayed at least one AMR phenotype, similar to the overall rate of AMR across all colonizing isolates (Table S8 online at doi.org/10.26188/14954931) (Data Set S2). Changes in AMR phenotypes within individual H. influenzae or *H. parainfluenzae* strains were observed during both persistent colonization (resistance phenotypes varied in 8/13 individuals, 62%) and transmission chains (resistance phenotypes varied in 4/6 transmission pairs, 67%), indicating short-term evolution of resistance (Data Set S2).

## DISCUSSION

H. influenzae and *H. parainfluenzae* colonization of the airways was strikingly common in this cohort, with >80% of participants contributing ≥1 Haemophilus culture-positive respiratory sample during the 1-year period of study (Table 1) and 58.5% contributing ≥2 such samples. The point prevalence was 4.6% for H. influenzae and 32.1% for *H. parainfluenzae*, and repeat colonization with *H. parainfluenzae* was much more common than that with H. influenzae (detected in 51% and 4.8% of participants, respectively). *H. parainfluenzae* also displayed a significantly higher frequency of AMR (Table 2), which was perhaps linked to its increased carriage rate. Globally, the H. influenzae carriage rate in children varies, likely due to differences in cohort demographics and geographical location. H. influenzae has been reported to be recoverable from the nasopharynx in 8% to 34% of children with CF (40, 48–52), consistent with H. influenzae carriage estimation in this CF cohort. The frequency of *H. parainfluenzae* airway colonization has not been established in children (with or without CF) despite the potential to opportunistically cause disease and act as a reservoir for AMR genes.

Substantial genetic diversity was observed for both H. influenzae and *H. parainfluenzae* isolates cultured from the airways of participants in this study (Fig. 1). Unsurprisingly, only two H. influenzae isolates (from a single patient) were predicted to be encapsulated (*cap-e*); they belonged to a known clonal capsule-positive lineage (ST18). The remaining H. influenzae isolates belong to the highly heterogenous NTHi group, similar to those detected in other studies examining nasopharyngeal colonization, which consistently report NTHi as the dominant H. influenzae subtype in the respiratory tract (40, 41).

An analysis of H. influenzae core-genome SNVs using phylogenetics and DAPC showed no apparent lineage associations with age group, specimen type, disease status, or geographical location (Fig. S4 and S5 online at doi.org/10.26188/14954931). This finding is consistent with prior studies of NTHi which reported finding no evidence for phylogenetic signals of geographical origin (53, 54) or clinical source (54, 55). However, whole-genome k-mer DAPC revealed distinct clustering of RCH CF isolates with others that were noninvasive, collected from the respiratory tract, isolated from children, and circulating in Australia (based on the respective individual discriminant functions, see Fig. 2). Hence, H. influenzae isolates of distinct epidemiological origins are differentiable based on variation in accessory genes but not by allelic variation in the core genome.

The structured variability across the accessory genome could potentially be explained by niche-specific positive selection of genes that confer increased fitness. For example, fixation of ICE*Hin*1056 in respiratory H. influenzae populations has been previously observed within 2 weeks of amoxicillin treatment but subsequently lost (or resistant strains outcompeted) 12 weeks after the initial treatment (56). The Hia and HMW adhesins, Hif pilus, and IgA proteases are H. influenzae virulence factors that are also differentially present in strains (53, 57) and play a role in the colonization of specific niches like the respiratory tract (58–61). Genome-wide association analysis could potentially identify other contributing factors (62); however, this identification is beyond the scope of the present study.

Antimicrobial therapy is used routinely both to control bacterial lung infections of CF patients (63) and also as an antimicrobial prophylaxis. Augmentin is routinely used for both of these purposes. Cotrimoxazole is used at many specialist CF centers, but it is not routine at RCH; however, resistance was still observed in nearly a one-third of H. influenzae and *H. parainfluenzae*. Regular use of antimicrobials is known to induce resistance, and indeed, we observed a high rate of AMR in isolates collected in this study, with resistance to one or more drugs observed in 52% H. influenzae and 82% *H. parainfluenzae* (Table 2). AMR rates in H. influenzae isolated from the respiratory tract in non-CF patients vary between studies, with recent reports of ampicillin resistance at 23.9% to 58.5%, augmentin at 0% to 10.4%, cefotaxime at 0% to 5.9%, cotrimoxazole at 51.2% to 71.1%, and rifampicin at 0% and 4.8% (18–27). Similar rates have been reported for *H. parainfluenzae* in non-CF patients, as follows: for ampicillin, 13.2% to 18.5%; augmentin, 0% to 12.5%; cefotaxime, 0% to 0.3%; cotrimoxazole, 14.9% to 44.2%; and rifampicin, 26.7% (28–30). AMR rates detected in the present study are mostly in line with rates in these reports, with the exception of higher rates of resistance in *H. parainfluenzae* versus H. influenzae. The only other report of such a difference is an earlier study in our setting (children with CF at RCH, 1998 to 2012) (31), which also found higher rates of resistance in *H. parainfluenzae* than in H. influenzae and showed that rates of ampicillin, augmentin, and cotrimoxazole resistance increased significantly in *H. parainfluenzae* over the 15-year duration of the study.

Most of the AMR phenotypes were explained by the presence of known genetic determinants. Ampicillin resistance was the most readily explained by known mechanisms, with all H. influenzae-resistant isolates and 67% of *H. parainfluenzae*-resistant isolates harboring the acquired gene *bla*_TEM_ or resistance-associated mutations in PBP genes (Data Set S2). The exceptions were augmentin and ceftriaxone; inhibitor-resistant *bla*_TEM_ alleles accounted for just 20% of augmentin resistance in *H. parainfluenzae* and none in H. influenzae, and no mechanisms for ceftriaxone resistance were identified.

Novel mutations in AMR-associated proteins discovered through association analysis increased the proportion of resistance explained by amino acid substitutions from 13.4% to 31.3%. Several mutations in FolA and FolP were associated with cotrimoxazole resistance (Table S5 and S6 online at doi.org/10.26188/14954931), including both novel and previously established mutations (46). Notably, we identified an insertion in *H. parainfluenzae* FolP that was strongly linked with cotrimoxazole resistance and shared the same location as the H. influenzae FolP-P64ins mutation, which has been demonstrated to induce sulfamethoxazole resistance (64). Mutations for rifampicin resistance were identified in H. influenzae (RpoB-A1131T) and *H. parainfluenzae* (RpoB-T724I); the latter was also recently reported as resistance associated in an independent study of *H. parainfluenzae* (19).

Consistent with previous studies, nearly all acquired genes detected here in Haemophilus isolates were localized to either an ICE*Hin* element (65, 66) or small *bla*_TEM_ plasmids (34, 44, 67). Novel variants of acquired resistant determinants were also observed, including a new ICE*Hin*-encoded *bla*_TEM_ allele associated with augmentin resistance in *H. parainfluenzae* and a novel plasmid harboring *bla*_TEM-1_.

Not all AMR could be explained by an underlying genetic component. This result is likely due in part to a lack of statistical power for detecting novel resistance-associated variants, even when taking a candidate-gene approach as we did, due to the small sample size. This limitation is particularly problematic for H. influenzae, for which only 24 sequenced isolates were available; for example, FolP-G189C was associated with cotrimoxazole resistance in both H. influenzae and *H. parainfluenzae* but was statistically significant only in *H. parainfluenzae* after adjustment for multiple testing (Table S5 and S6 online at doi.org/10.26188/14954931). Additionally, AMR phenotypes are not always reproducible, and AMR genes or mutations can be lost during subculture to extract DNA for sequencing. Moreover, it is conceivable that some single chromosomal mutations reported here are alone insufficient to confer resistance and instead may require a stepwise acquisition of additional mutations before resistance is gained.

A small number of isolates originally identified biochemically as H. influenzae were found to be *H. parainfluenzae* via WGS (*n* = 3) and vice versa (*n* = 5; Fig. S1 online at doi.org/10.26188/14954931). The definitive underlying cause of this discrepancy is unclear but could be explained by several possibilities, including the presence of both H. influenzae and *H. parainfluenzae* in the same sample or inaccuracies in the biochemical species identification test. The overall rate of discordance between biochemical and genomic species identification was ≤7%, suggesting that studies of H. influenzae colonization or infection that rely solely on biochemical identification without additional confirmation may suffer from both false positives and false negatives. There is little published data on the persistence of Haemophilus colonization in the lungs of children; however, there is some evidence that H. influenzae strain persistence is associated with chronic respiratory disease and does not occur in healthy childhood cohorts (68). This study reveals for the first time strain persistence of H. influenzae and *H. parainfluenzae* in the lungs of children with cystic fibrosis for up to 349 days and estimates the carriage rate of persistent strains in the cohort to be 15.6% (95% CI, 8.4% to 22.6%). Strain persistence likely arises due to substantial selective pressure exerted by extensive and prolonged administration of antimicrobials or niche adaptation to the diseased lung. For example, mutations in the single-strand mispairing mechanism allow H. influenzae to alter the expression of nutrient uptake systems and surface molecules, such as adhesins, during persistent colonization in adult patients with chronic obstructive pulmonary disorder (54). Other important CF pathogens, such as P. aeruginosa and members of the Burkholderia cepacia complex, have been shown to undergo similar changes to surface molecules and remodeling of regulatory networks during persistence (69–71). In addition to strain persistence, we observed that participants were frequently colonized by different strains of H. influenzae and *H. parainfluenzae* across clinic visits, indicating that Haemophilus colonization is a dynamic process and suggesting that strains of both species compete to occupy the niche.

There were six instances where participants shared the same Haemophilus strain. Three of the six cases were supported by epidemiological links whereby participants shared clinic visit days (Fig. S7 online at doi.org/10.26188/14954931), and the remaining three cases shared visit days with possible intermediaries (Fig. S7 online at doi.org/10.26188/14954931). Nosocomial transmission of CF pathogens, such as P. aeruginosa, has been demonstrated in other settings (71, 72) and historically at our center (73, 74), as has cross-infection with Mycobacterium abscessus (75). These findings have led to strict infection control practices in CF clinics such as RCH with strict isolation in both clinics and inpatient areas, wearing of face masks by patients in all public spaces, and strict gloving and gowning by all clinical staff. Notably, preceding the introduction of such stringent infection-control measures, sharing of RCH CF clinic visit days was common in our cohort, and nearly all participant pairs could be connected either through a shared clinic visit day (15%) or shared visit days with a single intermediary (72.4%). Hence, it is not clear whether the overlap in visit days could be circumstantial or strain sharing may reflect circulation of strains in the general community rather than nosocomial transmission. Future studies in settings where fewer patients share visit days may be better able to differentiate these possibilities.

This study provides the first insights into the population dynamics and genomic determinants of AMR among colonizing H. influenzae and *H. parainfluenzae* strains in a pediatric CF cohort and identifies multiple novel AMR determinants particularly for *H. parainfluenzae*. Notably, while relatively little attention has been paid to *H. parainfluenzae* colonization in children due to its relative lack of pathogenicity, our data indicate it is a common colonizer that can persist in the respiratory tract of CF children and is very frequently drug resistant. The high frequency of AMR in *H. parainfluenzae*, of which most was encoded in mobile elements that can transfer to H. influenzae, indicates that *H. parainfluenzae* could serve as a reservoir for the emergence and spread of AMR to H. influenzae which is of more significant clinical concern in children with and without CF. Further insights are essential and will inform antimicrobial treatment and stewardship in the future. Understanding the role of H. influenzae and *H. parainfluenzae* in early CF disease progression falls within the province of the AREST CF program goals, and additional studies will aim to assess and explore the specific risk factors associated with early lung colonization by these Haemophilus species.

## MATERIALS AND METHODS

### Participant recruitment and sample and data collection.

Participants in this study are a subset of those enrolled in the Australian Respiratory Early Surveillance Team for Cystic Fibrosis (AREST CF) birth cohort who meet the following inclusion criteria: diagnosed with CF, under 12 years of age, resident in catchment area, and presented to the RCH CF clinic between February 2016 to February 2017 (16). Respiratory samples (bronchoalveolar lavage [BAL] fluid, sputum, or cough swabs) were routinely collected from participants during regular visits and cultured on chocolate agar in the RCH microbiological diagnostics laboratory as previously described (16). During the 1-year study period, 847 samples collected from 147 study participants were analyzed and yielded 39 isolates identified as H. influenzae and 272 identified as *H. parainfluenzae* (identified using the X and V factor test). Isolates were tested for susceptibility to ampicillin (AMP), augmentin (AMC), cefotaxime (CTX), cotrimoxazole (STX), and rifampicin (RIF) using disk diffusion with CLSI breakpoints (Table S1 online at doi.org/10.26188/14954931).

### Bacterial isolates, sequencing, and assembly.

A total of 162 of the 311 Haemophilus isolates (*n* = 30, 77% of those biochemically identified as H. influenzae; and *n* = 132, 48.5% as *H. parainfluenzae*) were successfully resuscitated, subcultured, and transferred to the University of Melbourne for whole-genome sequencing (WGS). Isolates were plated onto chocolate agar and incubated at 37°C under microaerophilic conditions for 48 hours. Colonies were harvested and DNA extracted using GenFindV2 (Beckman Coulter), using proteinase K for bacterial lysis according to the manufacturer’s instructions. Short-read DNA libraries were prepared for all isolates with a Nextera XT kit (Illumina) and subsequently sequenced on the Illumina MiniSeq platform, generating paired-end reads of 151 bp each. DNA samples for long-read sequencing were prepared for a subset of 14 isolates using GenFindV2 (Beckman Coulter); a barcoded ligation library was prepared (SQK-LSK108, EXP-NBD103) and sequenced via an Oxford Nanopore MinION device on a R9.4.1 flow cell.

A total of 107 isolates (24 H. influenzae, 83 *H. parainfluenzae*) were successfully sequenced via Illumina and passed quality control, each yielding ≥150,000 high-quality reads (Fig. S1 online at doi.org/10.26188/14954931, and Data Set S1). Centrifuge v1.0.4b (76) was used to categorize isolates as either (i) pure H. influenzae or *H. parainfluenzae*, defined as one of these species at ≥50% relative abundance and the next most common species <20% relative abundance; (ii) contaminated H. influenzae or *H. parainfluenzae*, defined as one of these species at ≥50% relative abundance and a second species also highly represented (≥20% relative abundance); or (iii) other, where neither of these species exceeded 50% relative abundance (Fig. S1 online at doi.org/10.26188/14954931). Strain multiplicity for pure H. influenzae and *H. parainfluenzae* cultures was assessed by comparing the ratio of heterozygous to homozygous single nucleotide variant (SNV) calls (methods below) against an empirically determined threshold (H. influenzae, ≥0.025; or *H. parainfluenzae*, ≥0.100, calculated from public data sets); samples exceeding this threshold were considered mixed cultures and were excluded from further analysis (Fig. S1 online at doi.org/10.26188/14954931). Genomes were assembled with Unicycler v0.4.7 (77), using Illumina data in all cases and complemented by MinION data where available. All AMR plasmid sequences (listed in Table S4 online at doi.org/10.26188/14954931) were identified as circularized contigs in the assembly graphs. Read data and assemblies were deposited under the NCBI BioProject accession PRJNA668428 (see Data Set S1 for individual accessions).

### Population structure analysis.

The H. influenzae and *H. parainfluenzae* short-read Illumina data generated in this study (*n* = 107), and publicly available read sets for previously sequenced genomes of these species (*n* = 891; summarized in Table S2 online at doi.org/10.26188/14954931), were subjected to SNV detection, phylogenetic, and population structure analyses. SNVs (biallelic and polyallelic) were called using Bowtie2 v2.2.9 (for read mapping) and SAMtools v1.9 (for variant calling) via the RedDog pipeline v1beta.11 (https://github.com/katholt/reddog), using H. influenzae strain Rd KW20 (accession GCA_000027305.1) and *H. parainfluenzae* strain T3T1 (accession GCA_000210895.1) as reference genomes. For each species, core SNV alleles were defined as SNV alleles present in ≥95% genomes. Maximum likelihood conserved core-genome SNV phylogenies were inferred from alignments of core SNV alleles (263,940 [85.3% of all detected SNV] for 901 H. influenzae genomes and 329,046 [79.1% of all detected SNV] for 97 *H. parainfluenzae* genomes) using IQ-TREE v2.1.0 (78). Phylogenies were visualized with ggtree v1.14.6 (79) in R v3.5.2 (80). H. influenzae capsular serotype loci were detected from genome assemblies using hicap v1.0.0 (42), and sequence types (STs) were assigned to H. influenzae read sets using SRST2 v0.2.0 (81) with the H. influenzae multilocus sequence typing (MLST) database (82) (https://pubmlst.org/organisms/haemophilus-influenzae).

Discriminant analysis of principal components (DAPC) (83) was conducted to explore the relationship between bacterial population structure and sample source using k-mers (of length *k *= 16) extracted from assemblies. Frequencies of k-mers were counted in each assembly with fsm-lite v1.0 (https://github.com/nvalimak/fsm-lite), and a presence-absence matrix was constructed. Due to memory limitations, random sets of 500,000 k-mers were selected from the presence-absence matrix to use as input for DAPC, which was performed with the R package adegenet v2.1.1 (84) in triplicate using different random k-mer subsets to ensure stability of results (and additionally using the core-genome SNVs called from reads).

### Analysis of antimicrobial determinants.

RCH isolates were investigated for known and novel AMR determinants. Reads and assemblies were screened using SRST2 v0.2.0 and BLAST v2.7.1, respectively, to identify alleles of horizontally transferred AMR genes curated in the ARG-ANNOT database (85). Exact matches for translated *bla*_TEM_ gene sequences were identified in the NCBI AMR database with BLAST to infer the spectrum of activity and inhibitor resistance.

Mutations in chromosomally encoded antimicrobial target genes (*ftsI*, *folA*, *rpoB*, and *pbp* genes) (43, 44, 46, 64, 86–92) were also investigated. An exhaustive search for PBP genes present in the H. influenzae and *H. parainfluenzae* reference genomes was performed by aligning translated gene sequences to all curated PBP protein sequences available in the Swiss-Prot database (93) to identify those with ≥80% coverage and ≥70% identity (Table S3 online at doi.org/10.26188/14954931). Nucleotide sequences for target genes (*ftsI*, *folA*, *rpoB*, and *pbp* genes) were extracted from RCH isolate assemblies, and the translated amino acid sequences were aligned using MAFFT v7.407 (94). Each alignment position was compared to the consensus sequence for all isolates that were sensitive to the relevant antimicrobial. Positions that varied were tested for statistical association with the corresponding antimicrobial susceptibility phenotype (expressed as a binary variable, insensitive [I/R] versus sensitive [S]) using Fisher’s exact test and using linear mixed models (LMMs) to correct for population structure by including a genetic relatedness matrix calculated from the alignment of biallelic SNVs. LMMs were fitted with GEMMA v0.98.1 (95), and significance was assessed by the Wald test. The resulting *P* values were adjusted for multiple testing using Benjamini-Hochberg correction on a per-gene basis. Significant variants (*P < *0.05) are reported in Table S5 and S6 (available online at doi.org/10.26188/14954931), and the distribution of variants in isolates are detailed in Data Set S2.

### Identification of persistent and transmitted strains.

Strains were defined as groups of closely related isolates with a pairwise genetic distance of ≤20. They were identified initially using complete-linkage hierarchical clustering based on SNV distances derived from the global conserved core-genome alignment (Fig. S2 online at doi.org/10.26188/14954931). To capture isolate pairs with inflated SNV counts due to small numbers of recombination events, the SNV distance thresholds for strain definition were set to ≤2,000 for H. influenzae and ≤4,000 for *H. parainfluenzae* (Fig. S2A and C online at doi.org/10.26188/14954931). Precise pairwise SNV distances within each potential strain group were obtained by mapping all isolates to the best within-group genome assembly (lowest *N*_50_ value) using RedDog v1beta.11 as described above. Recombination blocks were identified by comparing pairwise SNV densities within discrete 4-kbp windows along the genome with the mean pairwise SNV count for all windows, using a binomial test and Bonferroni correction to account for multiple testing within each strain group (Fig. S3 online at doi.org/10.26188/14954931). Genetic distance between isolate pairs was then defined as nonrecombinant SNVs plus the number of recombinant blocks, and strains were defined as groups of isolates with pairwise genetic distance of ≤20 (Fig. S2B and D online at doi.org/10.26188/14954931).

### Data availability.

Read data and assemblies of H. influenzae and *H. parainfluenzae* isolates are available through NCBI BioProject under the accession PRJNA668428. Accessions for individual isolates are additionally listed in Data Set S1.

## References

[1] Andersen DH. 1938. Cystic fibrosis of the pancreas and its relation to celiac disease: a clinical and pathologic study. Am J Dis Child 56:344–399. doi:10.1001/archpedi.1938.01980140114013.

[2] Engelhardt JF, Yankaskas JR, Ernst SA, Yang Y, Marino CR, Boucher RC, Cohn JA, Wilson JM. 1992. Submucosal glands are the predominant site of CFTR expression in the human bronchus. Nat Genet 2:2:240–248. doi:10.1038/ng1192-240. 1285365

[3] Sagel SD, Sontag MK, Wagener JS, Kapsner RK, Osberg I, Accurso FJ. 2002. Induced sputum inflammatory measures correlate with lung function in children with cystic fibrosis. J Pediatr 141:811–817. doi:10.1067/mpd.2002.129847. 12461498

[4] Gangell C, Gard S, Douglas T, Park J, de Klerk N, Keil T, Brennan S, Ranganathan S, Robins-Browne R, Sly PD, AREST CF. 2011. Inflammatory responses to individual microorganisms in the lungs of children with cystic fibrosis. Clin Infect Dis 53:425–432. doi:10.1093/cid/cir399. 21844026

[5] Waters VJ, Kidd TJ, Canton R, Ekkelenkamp MB, Johansen HK, LiPuma JJ, Bell SC, Elborn JS, Flume PA, VanDevanter DR, Gilligan P, Antimicrobial Resistance International Working Group in Cystic Fibrosis. 2019. Reconciling antimicrobial susceptibility testing and clinical response in antimicrobial treatment of chronic cystic fibrosis lung infections. Clin Infect Dis 69:1812–1816. doi:10.1093/cid/ciz364. 31056660

[6] Kidd TJ, Canton R, Ekkelenkamp M, Johansen HK, Gilligan P, LiPuma JJ, Bell SC, Elborn JS, Flume PA, VanDevanter DR, Waters VJ, Antimicrobial Resistance in Cystic Fibrosis International Working Group. 2018. Defining antimicrobial resistance in cystic fibrosis. J Cyst Fibros 17:696–704. doi:10.1016/j.jcf.2018.08.014. 30266518

[7] Hahn A, Burrell A, Fanous H, Chaney H, Sami I, Perez GF, Koumbourlis AC, Freishtat RJ, Crandall KA. 2018. Antibiotic multidrug resistance in the cystic fibrosis airway microbiome is associated with decreased diversity. Heliyon 4:e00795. doi:10.1016/j.heliyon.2018.e00795. 30238064 PMC6143701

[8] Hauser AR, Jain M, Bar-Meir M, McColley SA. 2011. Clinical significance of microbial infection and adaptation in cystic fibrosis. Clin Microbiol Rev 24:29–70. doi:10.1128/CMR.00036-10. 21233507 PMC3021203

[9] Sly PD, Gangell CL, Chen L, Ware RS, Ranganathan S, Mott LS, Murray CP, Stick SM. 2013. Risk factors for bronchiectasis in children with cystic fibrosis. N Engl J Med 368:1963–1970. doi:10.1056/NEJMoa1301725. 23692169

[10] García-Rodríguez JA, Fresnadillo Martínez MJ. 2002. Dynamics of nasopharyngeal colonization by potential respiratory pathogens. J Antimicrob Chemother 50:59–73. doi:10.1093/jac/dkf506. 12556435

[11] Kosikowska U, Korona-Głowniak I, Niedzielski A, Malm A. 2015. Nasopharyngeal and adenoid colonization by Haemophilus influenzae and Haemophilus parainfluenzae in children undergoing adenoidectomy and the ability of bacterial isolates to biofilm production. Medicine (Baltimore) 94:e799. doi:10.1097/MD.0000000000000799. 25950686 PMC4602522

[12] Chen RV, Bradley JS. 1999. Haemophilus parainfluenzae sepsis in a very low birth weight premature infant: a case report and review of the literature. J Perinatol 19:315–317. doi:10.1038/sj.jp.7200078. 10685246

[13] Black CT, Kupferschmid JP, West KW, Grosfeld JL. 1988. Haemophilus parainfluenzae infections in children, with the report of a unique case. Rev Infect Dis 10:342–346. doi:10.1093/clinids/10.2.342. 3287563

[14] Alsuhaibani MA. 2019. Premature infant with Haemophilus parainfluenzae sepsis: case report and literature review. J Trop Pediatr 65:638–641. doi:10.1093/tropej/fmz010. 30892629

[15] Rayner RJ, Hiller EJ, Ispahani P, Baker M. 1990. Haemophilus infection in cystic fibrosis. Arch Dis Child 65:255–258. doi:10.1136/adc.65.3.255. 2185699 PMC1792300

[16] Sly PD, Brennan S, Gangell C, de Klerk N, Murray C, Mott L, Stick SM, Robinson PJ, Robertson CF, Ranganathan SC, Australian Respiratory Early Surveillance Team for Cystic Fibrosis (AREST-CF). 2009. Lung disease at diagnosis in infants with cystic fibrosis detected by newborn screening. Am J Respir Crit Care Med 180:146–152. doi:10.1164/rccm.200901-0069OC. 19372250

[17] Mott LS, Park J, Murray CP, Gangell CL, de Klerk NH, Robinson PJ, Robertson CF, Ranganathan SC, Sly PD, Stick SM, AREST CF. 2012. Progression of early structural lung disease in young children with cystic fibrosis assessed using CT. Thorax 67:509–516. doi:10.1136/thoraxjnl-2011-200912. 22201161

[18] Li J-P, Hua C-Z, Sun L-Y, Wang H-J, Chen Z-M, Shang S-Q. 2017. Epidemiological features and antibiotic resistance patterns of Haemophilus influenzae originating from respiratory tract and vaginal specimens in pediatric patients. J Pediatr Adolesc Gynecol 30:626–631. doi:10.1016/j.jpag.2017.06.002. 28629795

[19] Tchatchouang S, Nzouankeu A, Hong E, Terrade A, Denizon M, Deghmane A-E, Ndiang SMT, Pefura-Yone E-W, Beng VP, Njouom R, Fonkoua M-C, Taha M-K. 2020. Analysis of Haemophilus species in patients with respiratory tract infections in Yaoundé, Cameroon. Int J Infect Dis 100:12–20. doi:10.1016/j.ijid.2020.08.040. 32827751

[20] Zhang Z, Chen M, Yu Y, Pan S, Liu Y. 2019. Antimicrobial susceptibility among Streptococcus pneumoniae and Haemophilus influenzae collected globally between 2015 and 2017 as part of the Tigecycline Evaluation and Surveillance Trial (TEST). Infect Drug Resist 12:1209–1220. doi:10.2147/IDR.S203121. 31190909 PMC6524636

[21] Kiedrowska M, Kuch A, Żabicka D, Waśko I, Ronkiewicz P, Wasiak K, Bojarska K, Hryniewicz W, Skoczyńska A. 2017. β-Lactam resistance among Haemophilus influenzae isolates in Poland. J Glob Antimicrob Resist 11:161–166. doi:10.1016/j.jgar.2017.08.005. 28818575

[22] Wang H-J, Wang C-Q, Hua C-Z, Yu H, Zhang T, Zhang H, Wang S-F, Lin A-W, Cao Q, Huang W-C, Deng H-L, Cao S-C, Chen X-J. 2019. Antibiotic resistance profiles of Haemophilus influenzae isolates from children in 2016: a multicenter study in China. Can J Infect Dis Med Microbiol 2019:6456321. doi:10.1155/2019/6456321. 31485283 PMC6710757

[23] Fluit AC, Florijn A, Verhoef J, Milatovic D. 2005. Susceptibility of European β-lactamase-positive and -negative Haemophilus influenzae isolates from the periods 1997/1998 and 2002/2003. J Antimicrob Chemother 56:133–138. doi:10.1093/jac/dki167. 15917287

[24] Bae S, Lee J, Lee J, Kim E, Lee S, Yu J, Kang Y. 2010. Antimicrobial resistance in Haemophilus influenzae respiratory tract isolates in Korea: results of a nationwide acute respiratory infections surveillance. Antimicrob Agents Chemother 54:65–71. doi:10.1128/AAC.00966-09. 19884366 PMC2798543

[25] Shiro H, Sato Y, Toyonaga Y, Hanaki H, Sunakawa K. 2015. Nationwide survey of the development of drug resistance in the pediatric field in 2000–2001, 2004, 2007, 2010, and 2012: evaluation of the changes in drug sensitivity of Haemophilus influenzae and patients’ background factors. J Infect Chemother 21:247–256. doi:10.1016/j.jiac.2014.11.012. 25596977

[26] Semczuk K, Dzierzanowska-Fangrat K, Lopaciuk U, Gabinska E, Jozwiak P, Dzierzanowska D. 2004. Antimicrobial resistance of Streptococcus pneumoniae and Haemophilus influenzae isolated from children with community-acquired respiratory tract infections in Central Poland. Int J Antimicrob Agents 23:39–43. doi:10.1016/j.ijantimicag.2003.05.013. 14732312

[27] Daoud Z, Cocozaki A, Hakime N. 2006. Antimicrobial susceptibility patterns of Haemophilus influenzae and Streptococcus pneumoniae isolates in a Beirut general university hospital between 2000 and 2004. Clin Microbiol Infect 12:86–90. doi:10.1111/j.1469-0691.2005.01303.x. 16460553

[28] Soriano F, Granizo JJ, Coronel P, Gimeno M, Ródenas E, Gracia M, García C, Fernández-Roblas R, Esteban J, Gadea I. 2004. Antimicrobial susceptibility of Haemophilus influenzae, Haemophilus parainfluenzae and Moraxella catarrhalis isolated from adult patients with respiratory tract infections in four southern European countries. The ARISE project. Int J Antimicrob Agents 23:296–299. doi:10.1016/j.ijantimicag.2003.07.018. 15164972

[29] Sierra Y, González-Díaz A, Tubau F, Imaz A, Cubero M, Càmara J, Ayats J, Martí S, Ardanuy C. 2020. Emergence of multidrug resistance among Haemophilus parainfluenzae from respiratory and urogenital samples in Barcelona, Spain. Eur J Clin Microbiol Infect Dis 39:703–710. doi:10.1007/s10096-019-03774-x. 31828685

[30] Orden MB, Martínez-Ruiz R, Millán Pérez R. 2005. Haemophilus spp. antimicrobial susceptibility in Health Area 6 in Madrid, Spain (2000–2004). Rev Esp Quimioter 18:173–178. 16130040

[31] Ebbing R, Robertson CF, Robinson PJ. 2015. Haemophilus influenzae and Haemophilus parainfluenza in cystic fibrosis: 15 years experience. J Med Microbiol Diagnosis S5:004. doi:10.4172/2161-0703.S5-004.

[32] Tristram S, Jacobs MR, Appelbaum PC. 2007. Antimicrobial resistance in Haemophilus influenzae. Clin Microbiol Rev 20:368–389. doi:10.1128/CMR.00040-06. 17428889 PMC1865592

[33] Juhas M, Power PM, Harding RM, Ferguson DJ, Dimopoulou ID, Elamin AR, Mohd-Zain Z, Hood DW, Adegbola R, Erwin A, Smith A, Munson RS, Harrison A, Mansfield L, Bentley S, Crook DW. 2007. Sequence and functional analyses of Haemophilus spp. genomic islands. Genome Biol 8:R237. doi:10.1186/gb-2007-8-11-r237. 17996041 PMC2258188

[34] Tristram SG, Franks LR, Harvey GL. 2012. Sequences of small blaTEM-encoding plasmids in Haemophilus influenzae and description of variants falsely negative for blaTEM by PCR. J Antimicrob Chemother 67:2621–2625. doi:10.1093/jac/dks264. 22782486

[35] Tristram SG, Pitout MJ, Forward K, Campbell S, Nichols S, Davidson RJ. 2008. Characterization of extended-spectrum beta-lactamase-producing isolates of Haemophilus parainfluenzae. J Antimicrob Chemother 61:509–514. doi:10.1093/jac/dkm523. 18245789

[36] Pfeifer Y, Meisinger I, Brechtel K, Gröbner S. 2013. Emergence of a multidrug-resistant Haemophilus influenzae strain causing chronic pneumonia in a patient with common variable immunodeficiency. Microb Drug Resist 19:1–5. doi:10.1089/mdr.2012.0060. 23095085

[37] González-Díaz A, Tubau F, Pinto M, Sierra Y, Cubero M, Càmara J, Ayats J, Bajanca-Lavado P, Ardanuy C, Marti S. 2019. Identification of polysaccharide capsules among extensively drug-resistant genitourinary Haemophilus parainfluenzae isolates. Sci Rep 9:4481. doi:10.1038/s41598-019-40812-2. 30872664 PMC6418240

[38] Pittman M. 1931. Variation and type specificity in the bacterial species Hemophilus influenzae. J Exp Med 53:471–492. doi:10.1084/jem.53.4.471. 19869858 PMC2131978

[39] Peltola H. 2000. Worldwide Haemophilus influenzae type b disease at the beginning of the 21st century: global analysis of the disease burden 25 years after the use of the polysaccharide vaccine and a decade after the advent of conjugates. Clin Microbiol Rev 13:302–317. doi:10.1128/CMR.13.2.302. 10756001 PMC100154

[40] Cardines R, Giufrè M, Pompilio A, Fiscarelli E, Ricciotti G, Bonaventura GD, Cerquetti M. 2012. Haemophilus influenzae in children with cystic fibrosis: antimicrobial susceptibility, molecular epidemiology, distribution of adhesins and biofilm formation. Int J Med Microbiol 302:45–52. doi:10.1016/j.ijmm.2011.08.003. 22001303

[41] Román F, Cantón R, Pérez-Vázquez M, Baquero F, Campos J, 2004. Dynamics of long-term colonization of respiratory tract by Haemophilus influenzae in cystic fibrosis patients shows a marked increase in hypermutable strains. J Clin Microbiol 42:1450–1459. doi:10.1128/JCM.42.4.1450-1459.2004. 15070988 PMC387613

[42] Watts SC, Holt KE. 2019. hicap: in silico serotyping of the Haemophilus influenzae capsule locus. J Clin Microbiol 57:e00190-19. doi:10.1128/JCM.00190-19. 30944197 PMC6535587

[43] Dabernat H, Delmas C, Seguy M, Pelissier R, Faucon G, Bennamani S, Pasquier C. 2002. Diversity of beta-lactam resistance-conferring amino acid substitutions in penicillin-binding protein 3 of Haemophilus influenzae. Antimicrob Agents Chemother 46:2208–2218. doi:10.1128/AAC.46.7.2208-2218.2002. 12069976 PMC127296

[44] García-Cobos S, Arroyo M, Campos J, Pérez-Vázquez M, Aracil B, Cercenado E, Orden B, Lara N, Oteo J. 2013. Novel mechanisms of resistance to β-lactam antibiotics in Haemophilus parainfluenzae: β-lactamase-negative ampicillin resistance and inhibitor-resistant TEM β-lactamases. J Antimicrob Chemother 68:1054–1059. doi:10.1093/jac/dks525. 23335113

[45] Leflon-Guibout V, Heym B, Nicolas-Chanoine M-H. 2000. Updated sequence information and proposed nomenclature for blaTEM genes and their promoters. Antimicrob Agents Chemother 44:3232–3234. doi:10.1128/AAC.44.11.3232-3234.2000. 11036062 PMC101642

[46] Sierra Y, Tubau F, González-Díaz A, Carrera-Salinas A, Moleres J, Bajanca-Lavado P, Garmendia J, Domínguez MÁ, Ardanuy C, Martí S. 2020. Assessment of trimethoprim-sulfamethoxazole susceptibility testing methods for fastidious Haemophilus spp. Clin Microbiol Infect 26:944.e1–944.e7. doi:10.1016/j.cmi.2019.11.022. 31811916

[47] Cruchaga S, Pérez-Vázquez M, Román F, Campos J. 2003. Molecular basis of rifampicin resistance in Haemophilus influenzae. J Antimicrob Chemother 52:1011–1014. doi:10.1093/jac/dkh008. 14613947

[48] Armstrong DS, Grimwood K, Carlin JB, Carzino R, Olinsky A, Phenlan PD. 1996. Bronchoalveolar lavage or oropharyngeal cultures to identify lower respiratory pathogens in infants with cystic fibrosis. Pediatr Pulmonol 21:267–275. doi:10.1002/(SICI)1099-0496(199605)21:5<267::AID-PPUL1>3.0.CO;2-K. 8726151

[49] Rosenfeld M, Emerson J, Accurso F, Armstrong D, Castile R, Grimwood K, Hiatt P, McCoy K, McNamara S, Ramsey B, Wagener J. 1999. Diagnostic accuracy of oropharyngeal cultures in infants and young children with cystic fibrosis. Pediatr Pulmonol 28:321–328. doi:10.1002/(SICI)1099-0496(199911)28:5<321::AID-PPUL3>3.0.CO;2-V. 10536062

[50] Razvi S, Quittell L, Sewall A, Quinton H, Marshall B, Saiman L. 2009. Respiratory microbiology of patients with cystic fibrosis in the United States, 1995 to 2005. CHEST 136:1554–1560. doi:10.1378/chest.09-0132. 19505987

[51] Breuer O, Schultz A, Turkovic L, de Klerk N, Keil AD, Brennan S, Harrison J, Robertson C, Robinson PJ, Sly PD, Ranganathan S, Stick SM, Caudri D. 2019. Changing prevalence of lower airway infections in young children with cystic fibrosis. Am J Respir Crit Care Med 200:590–599. doi:10.1164/rccm.201810-1919OC. 30811949

[52] Rosenfeld M, Gibson RL, McNamara S, Emerson J, Burns JL, Castile R, Hiatt P, McCoy K, Wilson CB, Inglis A, Smith A, Martin TR, Ramsey BW. 2001. Early pulmonary infection, inflammation, and clinical outcomes in infants with cystic fibrosis. Pediatr Pulmonol 32:356–366. doi:10.1002/ppul.1144. 11596160

[53] De Chiara M, Hood D, Muzzi A, Pickard DJ, Perkins T, Pizza M, Dougan G, Rappuoli R, Moxon ER, Soriani M, Donati C. 2014. Genome sequencing of disease and carriage isolates of nontypeable Haemophilus influenzae identifies discrete population structure. Proc Natl Acad Sci USA 111:5439–5444. doi:10.1073/pnas.1403353111. 24706866 PMC3986186

[54] Pettigrew MM, Ahearn CP, Gent JF, Kong Y, Gallo MC, Munro JB, D'Mello A, Sethi S, Tettelin H, Murphy TF. 2018. Haemophilus influenzae genome evolution during persistence in the human airways in chronic obstructive pulmonary disease. Proc Natl Acad Sci USA 115:E3256–E3265. doi:10.1073/pnas.1719654115. 29555745 PMC5889651

[55] Kc R, Leong KWC, Harkness NM, Lachowicz J, Gautam SS, Cooley LA, McEwan B, Petrovski S, Karupiah G, O’Toole RFY. 2020. Whole-genome analyses reveal gene content differences between nontypeable Haemophilus influenzae isolates from chronic obstructive pulmonary disease compared to other clinical phenotypes. Microbial Genomics 6:e000405. doi:10.1099/mgen.0.000405. PMC7641420 32706329

[56] Chung A, Perera R, Brueggemann AB, Elamin AE, Harnden A, Mayon-White R, Smith S, Crook DW, Mant D. 2007. Effect of antibiotic prescribing on antibiotic resistance in individual children in primary care: prospective cohort study. BMJ 335:429. doi:10.1136/bmj.39274.647465.BE. 17656505 PMC1962897

[57] Ecevit IZ, McCrea KW, Pettigrew MM, Sen A, Marrs CF, Gilsdorf JR. 2004. Prevalence of the hifBC, hmw1A, hmw2A, hmwC, and hia genes in Haemophilus influenzae isolates. J Clin Microbiol 42:3065–3072. doi:10.1128/JCM.42.7.3065-3072.2004. 15243061 PMC446296

[58] St Geme JW, Falkow S, Barenkamp SJ. 1993. High-molecular-weight proteins of nontypable Haemophilus influenzae mediate attachment to human epithelial cells. Proc Natl Acad Sci USA 90:2875–2879. doi:10.1073/pnas.90.7.2875. 8464902 PMC46199

[59] Kubiet M, Ramphal R, Weber A, Smith A. 2000. Pilus-mediated adherence of Haemophilus influenzae to human respiratory mucins. Infect Immun 68:3362–3367. doi:10.1128/IAI.68.6.3362-3367.2000. 10816486 PMC97602

[60] Atack JM, Day CJ, Poole J, Brockman KL, Timms JRL, Winter LE, Haselhorst T, Bakaletz LO, Barenkamp SJ, Jennings MP. 2020. The non-typeable Haemophilus influenzae major adhesin Hia is a dual function lectin that binds to human-specific respiratory tract sialic acid glycan receptors. bioRxiv 10.1128/mBio.02714-20. PMC7642680 33144377

[61] Poulsen K, Reinholdt J, Kilian M. 1992. A comparative genetic study of serologically distinct Haemophilus influenzae type 1 immunoglobulin A1 proteases. J Bacteriol 174:2913–2921. doi:10.1128/jb.174.9.2913-2921.1992. 1373717 PMC205944

[62] Kc R, Leong KWC, Harkness NM, Lachowicz J, Gautam SS, Cooley LA, McEwan B, Petrovski S, Karupiah G, O’Toole RF. 2020. Whole-genome analyses reveal gene content differences between nontypeable Haemophilus influenzae isolates from chronic obstructive pulmonary disease compared to other clinical phenotypes. Microb Genom 6:mgen000405. doi:10.1099/mgen.0.000405. 32706329 PMC7641420

[63] Flume PA, Mogayzel PJ, Robinson KA, Goss CH, Rosenblatt RL, Kuhn RJ, Marshall BC, Clinical Practice Guidelines for Pulmonary Therapies Committee. 2009. Cystic fibrosis pulmonary guidelines. Am J Respir Crit Care Med 180:802–808. doi:10.1164/rccm.200812-1845PP. 19729669

[64] Enne VI, King A, Livermore DM, Hall LMC. 2002. Sulfonamide resistance in Haemophilus influenzae mediated by acquisition of sul2 or a short insertion in chromosomal folP. Antimicrob Agents Chemother 46:1934–1939. doi:10.1128/AAC.46.6.1934-1939.2002. 12019111 PMC127234

[65] Mohd-Zain Z, Turner SL, Cerdeño-Tárraga AM, Lilley AK, Inzana TJ, Duncan AJ, Harding RM, Hood DW, Peto TE, Crook DW. 2004. Transferable antibiotic resistance elements in Haemophilus influenzae share a common evolutionary origin with a diverse family of syntenic genomic islands. J Bacteriol 186:8114–8122. doi:10.1128/JB.186.23.8114-8122.2004. 15547285 PMC529066

[66] Gibson MK, Forsberg KJ, Dantas G. 2015. Improved annotation of antibiotic resistance determinants reveals microbial resistomes cluster by ecology. ISME J 9:207–216. doi:10.1038/ismej.2014.106. 25003965 PMC4274418

[67] Søndergaard A, San Millan A, Santos-Lopez A, Nielsen SM, Gonzalez-Zorn B, Nørskov-Lauritsen N. 2012. Molecular organization of small plasmids bearing blaTEM-1 and conferring resistance to β-lactams in Haemophilus influenzae. Antimicrob Agents Chemother 56:4958–4960. doi:10.1128/AAC.00408-12. 22733069 PMC3421876

[68] Puig C, Marti S, Fleites A, Trabazo R, Calatayud L, Liñares J, Ardanuy C. 2014. Oropharyngeal colonization by nontypeable Haemophilus influenzae among healthy children attending day care centers. Microb Drug Resist 20:450–455. doi:10.1089/mdr.2013.0186. 24716536

[69] Silva IN, Santos PM, Santos MR, Zlosnik JEA, Speert DP, Buskirk SW, Bruger EL, Waters CM, Cooper VS, Moreira LM. 2016. Long-term evolution of Burkholderia multivorans during a chronic cystic fibrosis infection reveals shifting forces of selection. mSystems 1:e00029-16. doi:10.1128/mSystems.00029-16. 27822534 PMC5069766

[70] Lieberman TD, Michel J-B, Aingaran M, Potter-Bynoe G, Roux D, Davis MR, Skurnik D, Leiby N, LiPuma JJ, Goldberg JB, McAdam AJ, Priebe GP, Kishony R. 2011. Parallel bacterial evolution within multiple patients identifies candidate pathogenicity genes. Nat Genet 43:1275–1280. doi:10.1038/ng.997. 22081229 PMC3245322

[71] Marvig RL, Sommer LM, Molin S, Johansen HK. 2015. Convergent evolution and adaptation of Pseudomonas aeruginosa within patients with cystic fibrosis. Nat Genet 47:57–64. doi:10.1038/ng.3148. 25401299

[72] Stapleton PJ, Izydorcyzk C, Clark S, Blanchard A, Wang PW, Yau Y, Waters V, Guttman DS. 2020. Pseudomonas aeruginosa strain sharing in early infection among children with cystic fibrosis. Clin Infect Dis 16:ciaa788. doi:10.1093/cid/ciaa788. PMC8563227 32544950

[73] Griffiths AL, Wurzel DF, Robinson PJ, Carzino R, Massie J. 2012. Australian epidemic strain pseudomonas (AES-1) declines further in a cohort segregated cystic fibrosis clinic. J Cyst Fibros 11:49–52. doi:10.1016/j.jcf.2011.08.005. 21907639

[74] Armstrong DS, Nixon GM, Carzino R, Bigham A, Carlin JB, Robins-Browne RM, Grimwood K. 2002. Detection of a widespread clone of Pseudomonas aeruginosa in a pediatric cystic fibrosis clinic. Am J Respir Crit Care Med 166:983–987. doi:10.1164/rccm.200204-269OC. 12359658

[75] Yan J, Kevat A, Martinez E, Teese N, Johnson K, Ranganathan S, Harrison J, Massie J, Daley A. 2020. Investigating transmission of Mycobacterium abscessus amongst children in an Australian cystic fibrosis centre. J Cyst Fibros 19:219–224. doi:10.1016/j.jcf.2019.02.011. 30853372

[76] Kim D, Song L, Breitwieser FP, Salzberg SL. 2016. Centrifuge: rapid and sensitive classification of metagenomic sequences. Genome Res 26:1721–1729. doi:10.1101/gr.210641.116. 27852649 PMC5131823

[77] Wick RR, Judd LM, Gorrie CL, Holt KE. 2017. Unicycler: resolving bacterial genome assemblies from short and long sequencing reads. PLoS Comput Biol 13:e1005595. doi:10.1371/journal.pcbi.1005595. 28594827 PMC5481147

[78] Minh BQ, Schmidt HA, Chernomor O, Schrempf D, Woodhams MD, von Haeseler A, Lanfear R. 2020. IQ-TREE 2: new models and efficient methods for phylogenetic inference in the genomic era. Mol Biol Evol 37:1530–1534. doi:10.1093/molbev/msaa015. 32011700 PMC7182206

[79] Yu G, Smith DK, Zhu H, Guan Y, Lam TT-Y. 2017. ggtree: an r package for visualization and annotation of phylogenetic trees with their covariates and other associated data. Methods Ecol Evol 8:28–36. doi:10.1111/2041-210X.12628.

[80] R Core Team. 2019. R: a language and environment for statistical computing. R Foundation for Statistical Computing, Vienna, Austria.

[81] Inouye M, Dashnow H, Raven L-A, Schultz MB, Pope BJ, Tomita T, Zobel J, Holt KE. 2014. SRST2: rapid genomic surveillance for public health and hospital microbiology labs. Genome Med 6:90. doi:10.1186/s13073-014-0090-6. 25422674 PMC4237778

[82] Meats E, Feil EJ, Stringer S, Cody AJ, Goldstein R, Kroll JS, Popovic T, Spratt BG. 2003. Characterization of encapsulated and noncapsulated Haemophilus influenzae and determination of phylogenetic relationships by multilocus sequence typing. J Clin Microbiol 41:1623–1636. doi:10.1128/JCM.41.4.1623-1636.2003. 12682154 PMC153921

[83] Jombart T, Devillard S, Balloux F. 2010. Discriminant analysis of principal components: a new method for the analysis of genetically structured populations. BMC Genet 11:94. doi:10.1186/1471-2156-11-94. 20950446 PMC2973851

[84] Jombart T. 2008. adegenet: a R package for the multivariate analysis of genetic markers. Bioinformatics 24:1403–1405. doi:10.1093/bioinformatics/btn129. 18397895

[85] Gupta SK, Padmanabhan BR, Diene SM, Lopez-Rojas R, Kempf M, Landraud L, Rolain J-M. 2014. ARG-ANNOT, a new bioinformatic tool to discover antibiotic resistance genes in bacterial genomes. Antimicrob Agents Chemother 58:212–220. doi:10.1128/AAC.01310-13. 24145532 PMC3910750

[86] Groot R, de Sluijter M, Bruyn A, de Campos J, Goessens WH, Smith AL, Hermans PW. 1996. Genetic characterization of trimethoprim resistance in Haemophilus influenzae. Antimicrob Agents Chemother 40:2131–2136. doi:10.1128/AAC.40.9.2131. 8878594 PMC163486

[87] Mohd-Zain Z, Kamsani NH, Ahmad N. 2013. Molecular insights of co-trimoxazole resistance genes in Haemophilus influenzae isolated in Malaysia. Trop Biomed 30:584–590. 24522126

[88] Sanbongi Y, Suzuki T, Osaki Y, Senju N, Ida T, Ubukata K. 2006. Molecular evolution of β-lactam-resistant Haemophilus influenzae: 9-year surveillance of penicillin-binding protein 3 mutations in isolates from Japan. Antimicrob Agents Chemother 50:2487–2492. doi:10.1128/AAC.01316-05. 16801430 PMC1489784

[89] Wienholtz NH, Barut A, Nørskov-Lauritsen N. 2017. Substitutions in PBP3 confer resistance to both ampicillin and extended-spectrum cephalosporins in Haemophilus parainfluenzae as revealed by site-directed mutagenesis and gene recombinants. J Antimicrob Chemother 72:2544–2547. doi:10.1093/jac/dkx157. 28582518

[90] Ubukata K, Shibasaki Y, Yamamoto K, Chiba N, Hasegawa K, Takeuchi Y, Sunakawa K, Inoue M, Konno M. 2001. Association of amino acid substitutions in penicillin-binding protein 3 with β-lactam resistance in β-lactamase-negative ampicillin-resistant Haemophilus influenzae. Antimicrob Agents Chemother 45:1693–1699. doi:10.1128/AAC.45.6.1693-1699.2001. 11353613 PMC90533

[91] Kubota T, Higa F, Kusano N, Nakasone I, Haranage S, Tateyama M, Yamane N, Fujita J. 2006. Genetic analyses of beta-lactamase negative ampicillin-resistant strains of Haemophilus influenzae isolated in Okinawa, Japan. Jpn J Infect Dis 59:36–41. 16495632

[92] Mizoguchi A, Hitomi S. 2019. Cefotaxime-non-susceptibility of Haemophilus influenzae induced by additional amino acid substitutions of G555E and Y557H in altered penicillin-binding protein 3. J Infect Chemother 25:509–513. doi:10.1016/j.jiac.2019.02.010. 30879978

[93] Boutet E, Lieberherr D, Tognolli M, Schneider M, Bairoch A. 2007. UniProtKB/Swiss-Prot. Methods Mol Biol 406:89–112. doi:10.1007/978-1-59745-535-0_4. 18287689

[94] Katoh K, Standley DM. 2013. MAFFT multiple sequence alignment software version 7: improvements in performance and usability. Mol Biol Evol 30:772–780. doi:10.1093/molbev/mst010. 23329690 PMC3603318

[95] Zhou X, Stephens M. 2012. Genome-wide efficient mixed-model analysis for association studies. Nat Genet 44:821–824. doi:10.1038/ng.2310. 22706312 PMC3386377

